# Multi-omics insights into mosquito insecticide resistance for integrated vector management

**DOI:** 10.7717/peerj.21083

**Published:** 2026-04-20

**Authors:** Jie Li, Qiao-Yan Wang

**Affiliations:** Jiading District Center for Disease Control and Prevention, Jiading District Health Supervision Institute, Shanghai, China

**Keywords:** Mosquito vectors, Insecticide resistance, Multi-omics, Mechanisms, Precision control

## Abstract

Escalating insecticide resistance in mosquito vectors threatens the durability of vector-borne disease control and increasingly constrains the effectiveness of core interventions. This resistance is a multilayered adaptive phenotype arising from the combined action of target-site substitutions that reduce insecticide sensitivity, transcriptional and enzymatic upregulation of detoxification systems that enhance xenobiotic metabolism, cuticular and behavioral changes that limit exposure and penetration, and transporter-mediated efflux, with additional modulation by microbiota and local environmental conditions that shape phenotypic expression in the field. Current integrated vector management (IVM) strategies aim to mitigate resistance through operationally guided deployment of dual-active-ingredient or synergist-treated nets, indoor residual spraying with rotations or mixtures, integration of larval source management and habitat modification, and incorporation of nonchemical tools such as Wolbachia releases and genetic control, supported by routine resistance surveillance. However, much of the existing evidence remains fragmented, with an overreliance on a narrow set of insecticide classes and a limited number of genetic markers, variable phenotyping and performance metrics across settings, and insufficient prospective linkage between molecular signals and intervention impact under real transmission ecologies. Multi-omics frameworks provide a route to move beyond single-locus screening toward network-level reconstruction of resistance biology, enabling discovery of predictive biomarkers, pathway signatures, and metabolic readouts that can be translated into actionable diagnostics and locally optimized decision rules. Looking forward, omics-enabled precision surveillance integrated with field-deployable assays, standardized benchmarks, and model-informed adaptive management could support closed-loop resistance mitigation in which operational choices are continuously refined to preserve long-term intervention efficacy within IVM programs.

## Introduction

Large-scale insecticide use remains central to mosquito-borne disease control, with insecticide-treated nets and indoor residual spraying forming the operational backbone of national programs (Franklinos et al., 2019). Concurrently, arboviral activity has expanded. Consolidated analyses indicate a record 14.1 million reported dengue cases and 9,508 deaths worldwide in 2024, underscoring an ongoing multi-regional surge (Poespoprodjo et al., 2023). Beyond dengue, additional Aedes- and Culex-borne viruses continue to strain health systems: chikungunya case counts in the Americas exceeded 410,000 in 2023 with hundreds of deaths (Witte et al., 2024), West Nile virus activity rose above historical baselines in parts of Europe with record locally acquired cases in 2024 (Xie, Jiao & Sun, 2025; Bourgeois et al., 2025). Japanese encephalitis remains the leading cause of viral encephalitis across much of Asia with an estimated 100,000 clinical cases annually despite vaccine availability (Bharadwaj et al., 2025), and Zika persists at low levels with periodic focal outbreaks (Zinszer & Talisuna, 2023). Lymphatic filariasis also continues to place large populations at risk in settings where vector control interfaces with mass drug administration. Together, these patterns highlight both the indispensability of insecticides for public health impact and the vulnerability of current strategies to biological threats.

**Figure 1:**
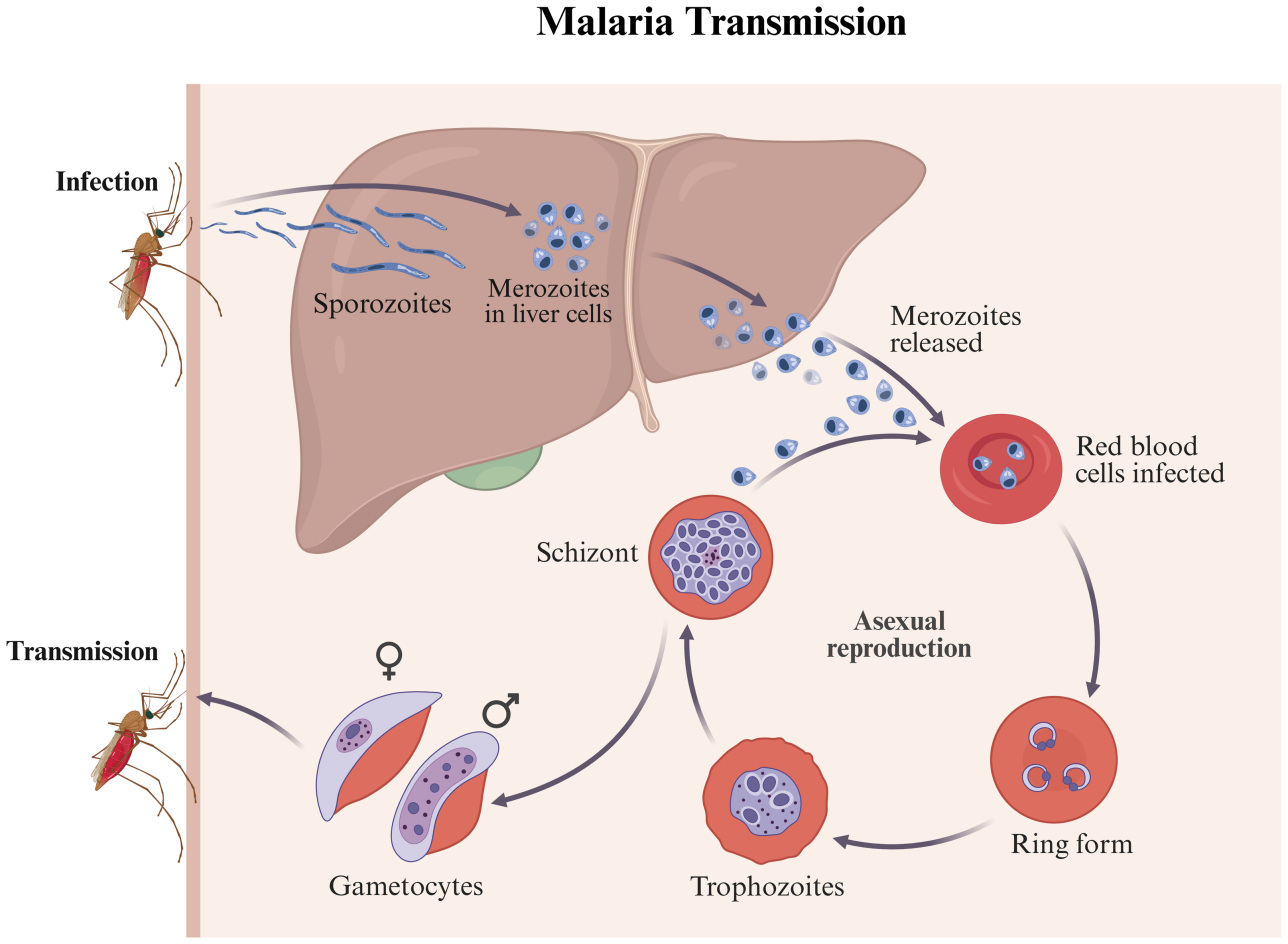
Malaria transmission and intra-host developmental cycle. During an infectious bite, sporozoites enter the bloodstream and invade hepatocytes to form schizonts, which release merozoites that infect red blood cells. The asexual blood-stage cycle (ring → trophozoite → schizont) causes erythrocyte rupture and symptoms; a subset differentiates into male/female gametocytes, which are taken up by mosquitoes to continue transmission. Arrows indicate developmental and transmission flow. This figure was created by BioRender (https://www.biorender.com/) and does not involve any copyright issues.

Within this context, the emergence and spread of insecticide resistance in mosquito populations pose a pervasive constraint on program sustainability (Niklas et al., 2023; Lehane et al., 2024). WHO compilations show that among 88 malaria-endemic countries reporting resistance data between 2010 and 2020, 78 detected resistance to at least one insecticide class and 29 reported resistance across all four major classes, underscoring the breadth of the problem in Anopheles vectors (Smith, Kasai & Scott, 2016). Similar pressures are increasingly documented in *Aedes* and *Culex*, with implications for arboviral control (Soria et al., 2024) (Fig. 1).

Mechanistically, mosquito insecticide resistance is increasingly recognized as a composite phenotype produced by interacting molecular and physiological layers rather than a single mutation. Target-site resistance is supported by well-characterized substitutions in the voltage-gated sodium channel, including the canonical kdr variants L1014F/L1014S, as well as acetylcholinesterase changes such as G119S that reduce sensitivity to organophosphates and carbamates, with field surveys and genotype–phenotype analyses repeatedly linking these alleles to elevated survival in standard bioassays (Koekemoer et al., 2011). Metabolic resistance contributes additional breadth through the induction and expansion of detoxification capacity, where overexpression and functional activity of cytochrome P450 monooxygenases, glutathione S-transferases, and carboxylesterases have been associated with multi-class resistance and cross-resistance patterns; importantly, synergist assays and enzyme-activity measurements provide causal support that enhanced xenobiotic metabolism can restore susceptibility when detox pathways are inhibited (Nauen et al., 2022; Katsavou et al., 2022). Beyond these internal mechanisms, reduced penetration *via* cuticular remodeling and altered cuticle composition can lower the delivered dose, while transporter-mediated efflux and behavioral avoidance further decrease effective exposure to treated surfaces, helping explain why phenotypic resistance often exceeds what is predicted by target-site genotyping alone (Djiappi-Tchamen et al., 2021; Alout et al., 2017). Collectively, this multilayer architecture offers a mechanistic basis for why resistance can emerge rapidly, persist under heterogeneous selection, and vary across ecological contexts, thereby motivating surveillance approaches that integrate genetic markers with transcriptomic, proteomic, and metabolomic signatures to better predict operational failure.

Multi-omics investigations indicate that these phenotypes often arise from convergent processes involving gene-expression remodeling, copy-number variation, and pathway-level reprogramming rather than single-locus effects, reinforcing the limitations of solitary molecular markers as universal predictors of field risk (Katsuta et al., 2019; Nakao & Banba, 2016). Early field signals for newer chemistries, for example clothianidin, emphasize the need for proactive susceptibility thresholds and stratified responses as local patterns shift. Complementary non-chemical tools have accrued robust evidence; randomized deployment of wMel Wolbachia-infected *Aedes aegypti*, for instance, reduced virologically confirmed dengue by approximately 77% and hospitalizations by 86% in Indonesia, strengthening the rationale for integrated, multi-tool approaches (Utarini et al., 2021; Ross et al., 2022).

Against this backdrop, a precision-oriented framework for resistance management centers on rigorous phenotypic testing, curated molecular markers, and multi-omics profiling to construct dynamic risk landscapes that inform the design and timing of interventions. At the operational level, evidence supports optimization of tool combinations that integrate dual-active-ingredient nets, indoor residual spraying rotations and mixtures, synergist formulations, and insecticides with novel modes of action, together with Wolbachia deployment, environmental management, and source reduction. Modeling, economic evaluation, and adaptive deployment link surveillance to response in a feedback-driven system in which data streamline decisions and outcomes iteratively refine strategy. Such integration is essential to slow the evolution of resistance while preserving long-term effectiveness.

## Survey methodology

We conducted a structured scoping review consistent with PRISMA-ScR guidance. Literature searches were performed in PubMed/MEDLINE, Embase, Web of Science Core Collection, and Scopus for records published from January 2000 to September 2025. Searches were complemented by grey literature from The World Health Organization (WHO), Centers for Disease Control (CDC), and Innovative Vector Control Consortium (IVCC), as well as trial registries and programme reports relevant to operational vector control. Search strings combined controlled vocabulary and free-text terms for mosquito genera (*Aedes*, *Anopheles*, *Culex*), insecticide classes (*e.g.*, pyrethroids, organophosphates, carbamates, neonicotinoids, and newer chemistries such as clothianidin), resistance mechanisms (*e.g.*, *kdr*, *Ace-1*, cytochrome P450s, Glutathione S-transferases (GSTs), carboxylesterases, cuticular modification, efflux, behavioural avoidance), omics modalities (genomics, transcriptomics, proteomics, metabolomics, single-cell and spatial), field-deployable diagnostics and surveillance platforms, intervention combinations (dual-active-ingredient nets, indoor residual insecticide spraying (IRS) rotations/mixtures, synergist formulations, Wolbachia releases, environmental/source reduction, genetic control), and links to transmission modelling and economic evaluation.

### The intended audience

This review targets a multidisciplinary readership involved in mosquito-borne disease control and resistance management. Primary audiences include entomologists and vector biologists; national and sub-national vector-control programme managers; public-health policymakers and procurement partners; molecular epidemiologists and bioinformaticians developing resistance diagnostics; product developers of new chemistries, synergists, and field-deployable assays; infectious-disease modellers and health economists; and implementation scientists and donors seeking to link surveillance outputs to operational decisions within integrated vector management.

## Molecular and Phenotypic Mechanisms of Mosquito Insecticide Resistance

**Figure 2:**
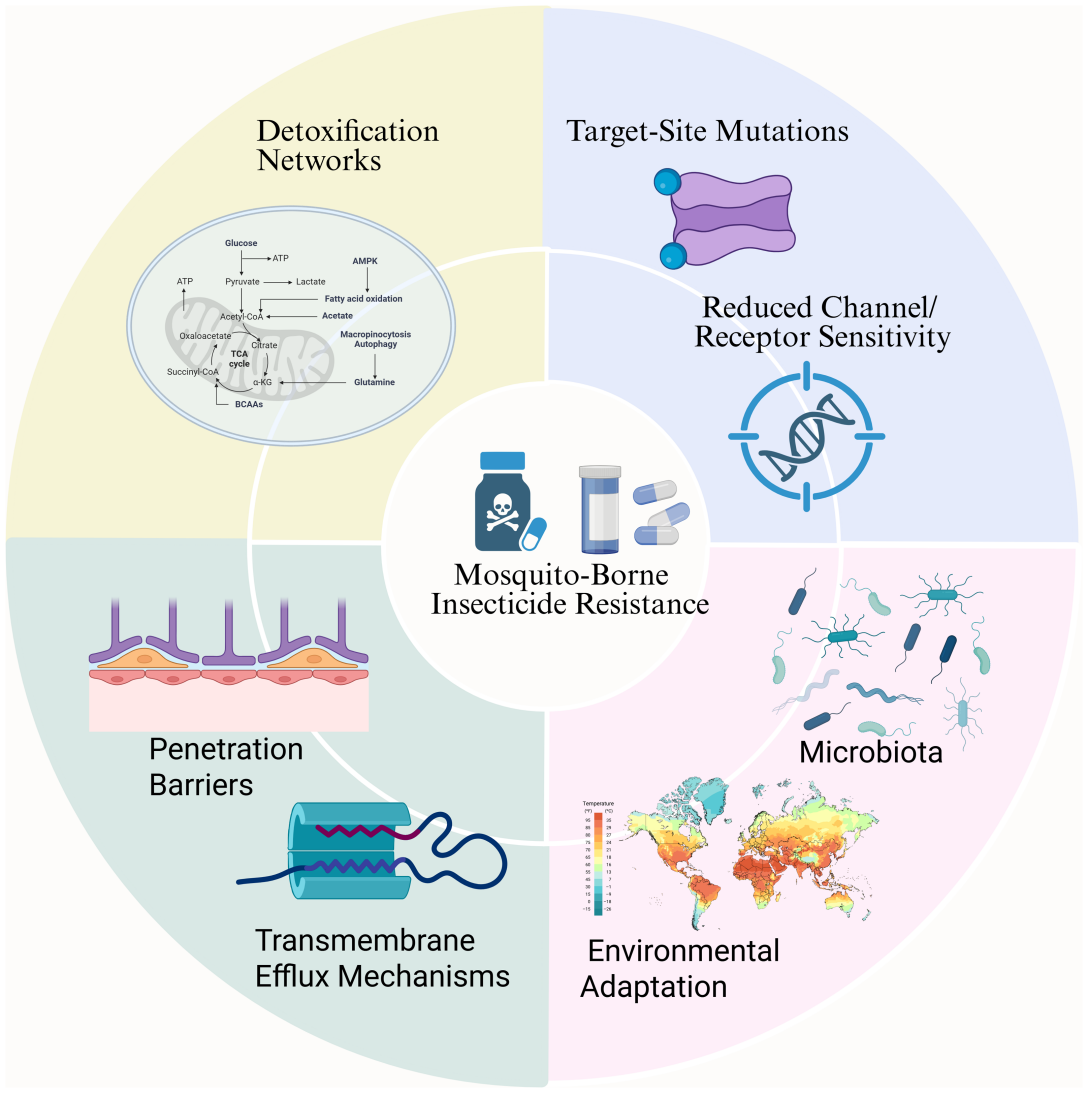
Multilayered mechanisms underlying mosquito insecticide resistance. Selection pressure from insecticide exposure. Outer sectors summarize: (1) Target-site mutations with reduced channel/receptor sensitivity (*e.g.*, VGSC *kdr* L1014F/S; Ace-1 G119S); (2) Detoxification network reprogramming (upregulated P450s, GSTs, CCEs, UGTs); (3) Penetration barriers (cuticle/CHC changes) and (4) Transmembrane efflux (ABC transporters/P-gp); (5) Microbiota effects; and (6) Environmental adaptation (thermal/chemical stress–induced cross-tolerance). These layers interact to increase survival and drive population-level resistance. This figure was created by BioRender (https://www.biorender.com/) and does not involve any copyright issues.

The development of insecticide resistance in mosquito populations involves a complex network of biological mechanisms operating at multiple levels, from molecular mutations to population-wide adaptations. Each layer contributes to increased survival probability in the face of chemical interventions. This section reviews the major mechanistic categories currently recognized in resistance research (Fig. 2).

### Target-site mutations and reduced channel/receptor sensitivity

In public health vector control, pyrethroids have received disproportionate attention because they have been used extensively in long lasting insecticidal nets for decades; however, mosquito insecticide resistance is not confined to this class. World Health Organization surveillance syntheses indicate that, in addition to pyrethroid and DDT related resistance, resistance to organophosphates and carbamates has been repeatedly documented across multiple regions and has shown increasing trends in several settings, underscoring that real world resistance landscapes are shaped by coexisting selective pressures from multiple insecticide classes rather than a single category (Grau-Bové et al., 2021). This point is particularly consequential for indoor residual spraying practice, because under widespread pyrethroid resistance, organophosphates such as pirimiphos methyl have been adopted as key alternatives, and their field performance and long-term durability depend critically on concurrent monitoring and management of the corresponding resistance pathways. Accordingly, systematic coverage of non-pyrethroid resistance in this review is essential for translating mechanistic evidence into operationally actionable integrated vector management strategies.

Organophosphates and carbamates share a primary target in acetylcholinesterase, and resistance to these compounds can arise through both target site modifications and enhanced metabolic clearance. At the target site level, the Ace1 G119S substitution has been widely associated with resistance to organophosphates and carbamates, and in natural populations it is frequently accompanied by gene duplication or copy number expansion that can mitigate fitness costs, thereby facilitating long term persistence and spread under multi-insecticide selection regimes (Assogba et al., 2015). The genetic basis of pirimiphos methyl resistance has also been investigated within population genomics frameworks, where signals of selection and population structure analyses have been used to infer evolutionary origins and dissemination patterns, indicating that organophosphate resistance is not inherently stable after class substitution and instead requires sustained molecular surveillance and adaptive management. In parallel, phenotypic resistance to the carbamate bendiocarb has been repeatedly observed in multiple African settings, yet its mechanistic basis is not always explained by Ace1 variation alone (Aïkpon et al., 2013). Evidence from some regions suggests that metabolic mechanisms, including upregulation of multiple P450 genes, may play a dominant role. Collectively, these findings emphasize that non pyrethroid resistance is mechanistically diverse and geographically contingent, and cannot be reliably inferred from a single marker.

For organochlorines, although DDT and dieldrin have been discontinued for routine use in many regions, their resistance pathways exhibit a pronounced legacy effect that can still influence interpretations of cross resistance risk and molecular surveillance. For dieldrin in particular, A296S and A296G substitutions in the Rdl locus encoding the GABA receptor have been linked to resistance and show evidence of strong selection and interspecific introgression in African anophelines, consistent with long term maintenance and spread on epidemiologically relevant timescales (Wondji et al., 2011). Notably, rdl associated variants can remain detectable in vector populations years after discontinuation, implying that historical insecticide use and contemporary cross selection should be considered jointly within an integrated analytical framework (Portwood et al., 2022). Incorporating organochlorine associated target site resistance into a consolidated resistance landscape therefore helps avoid overlooking persistent genetic backgrounds that may shape present day responses to interventions.

Mosquito resistance in operational settings commonly reflects concurrent resistance across insecticide classes and the coupling of multiple mechanisms. Consequently, replacing pyrethroids with organophosphates or carbamates in IRS does not eliminate resistance risk, but rather shifts selection pressure toward Ace1 related pathways and their associated metabolic networks.

#### Voltage-gated sodium channel

Target-site resistance is one of the most direct and well-characterized mechanisms, where point mutations occur in the genes encoding insecticide target proteins, such as ion channels or key enzymes, resulting in reduced binding affinity or diminished functional response to the insecticide. Reduced sensitivity of Nav underlies much of the failure of DDT and pyrethroids. Amino-acid substitutions cluster in S6 segments and nearby linkers and either destabilize the pyrethroid-stabilized open state or impede ligand access. In *Anopheles* and *Culex*, IIS6 codon 1014 substitutions (L1014F or L1014S) recur under strong selection. In *Aedes aegypti*, broader constellations are common, including V1016G or V1016I, S989P, F1534C or F1534L, V410L and T1520I, with double or triple haplotypes (for example S989P+V1016G or S989P+V1016G+F1534C) conferring higher resistance and a wider pyrethroid spectrum (Menze et al., 2016). Recent population and experimental work shows that kdr genotype composition predicts resistance strength and can influence intervention performance

#### Acetylcholinesterase insensitivity

Organophosphates and carbamates act by inhibiting synaptic Acetylcholinesterase (AChE). Point mutations in *ace-1* or *ace-2* reduce inhibitor affinity while retaining catalytic throughput. Three substitutions are functionally supported in mosquitoes: G119S in the oxyanion hole introduces steric hindrance to the phosphylated intermediate (Temeyer et al., 2025). F290V reshapes the acyl pocket; F331W perturbs substrate guidance and binding. These variants are prominent in *Culex pipiens* and *Anopheles gambiae*, with more heterogeneous evidence in *Ae. aegypti* and *An. stephensi*. Recombinant and biochemical assays consistently show elevated residual AChE activity in mutant backgrounds (Alout et al., 2007; Carlier et al., 2017).

#### GABA-gated chloride channel variants

Cyclodienes and fipronil target the GABA-gated chloride channel (RDL) subunit of the GABA receptor. Substitutions at the pore-lining M2 helix, notably A296G or A296S in *Aedes*, alter channel pharmacology and reduce antagonist potency. The *rdl* locus is further diversified by alternative splicing and extensive RNA A-to-I editing, generating isoforms with distinct kinetics and drug sensitivity. Species-specific editing patterns have been documented across major mosquito vectors and can modulate both GABA and insecticide responses, with some variants carrying fitness costs *via* altered neuronal signaling (Taylor-Wells et al., 2018). Notably, parallel evolution has been observed across species, with functionally equivalent mutations such as *kdr* and *Rdl* (resistance to dieldrin) arising independently in multiple insect taxa (Castro-Janer et al., 2021).

#### Structural variants and dosage effects at target loci

Target-site resistance is not restricted to single-nucleotide substitutions. Copy-number changes at *ace-1* create heterogeneous or homogeneous duplications that increase enzyme dosage and can buffer fitness penalties, stabilizing resistance under fluctuating selection. Recent genomic work in *An. gambiae* sensu lato clarifies the origin, maintenance and adaptive value of these duplicated alleles and provides diagnostics to track them in surveillance (Claret et al., 2024). While target-site mutations are often linked to strong resistance phenotypes, they may impose fitness costs that require evolutionary trade-offs between resistance and survival under natural conditions (Platt et al., 2015). In addition to single-nucleotide mutations, copy number variations (CNVs) can also influence target-site resistance. For example, duplications of the Ace-1 gene have been reported in some mosquito populations, leading to overexpression of the enzyme and enhanced resistance (Diallo et al., 2022).

Overall, target-site mutations directly interfere with the “lock-and-key” interaction between insecticides and their molecular targets, representing a primary and frequently observed driver of resistance evolution.

### Penetration barriers and transmembrane efflux mechanisms

Penetration resistance refers to a type of insecticide resistance whereby insects reduce the absorption of insecticides through the body surface, commonly referred to as cuticular resistance (Meng et al., 2023). Alterations in the composition or structure of the insect cuticle, particularly its wax and chitin layers, can significantly influence the rate and extent of insecticide penetration. One key factor is the change in the composition and quantity of cuticular hydrocarbons (CHCs) (Moore et al., 2022; Caselli et al., 2023). In resistant strains of *Anopheles* mosquitoes, cytochrome P450 enzymes of the CYP4G subfamily, responsible for the biosynthesis of long-chain hydrocarbons, undergo functional changes that result in a denser or chemically altered wax layer (Balabanidou et al., 2016; Fagbohun et al., 2019). This modification reduces the permeability of insecticides such as deltamethrin and cyhalothrin.

For example, overexpression of the CYP4G16 gene in *Anopheles gambiae* leads to the accumulation of wax hydrocarbons, significantly decreasing insecticide penetration across the cuticle (Kouadio et al., 2023). Similar thickening or hardening of the cuticle has been observed in other resistant insect species, suggesting this is a widespread and conserved mechanism.

In addition to passive reduction in penetration, active transporters also play a critical role in resistance. ATP-binding cassette (ABC) transporters are a large family of transmembrane proteins that utilize ATP hydrolysis to pump a wide range of substrates, including toxins and insecticides, out of cells (Wu et al., 2019a). Among them, P-glycoprotein (P-gp), classified under the ABCB subfamily, has been widely associated with multidrug resistance. In mosquitoes, P-gp is believed to actively expel insecticides or their metabolites, thereby reducing their intracellular concentrations and toxic effects (Buss, McCaffery & Callaghan, 2002; Huang et al., 2024).

Experimental data have shown that silencing P-gp expression in *Aedes aegypti* larvae significantly increases their susceptibility to the organophosphate temephos, with mortality rates rising by approximately 57 percent (Figueira-Mansur et al., 2013). This finding supports the role of P-gp in reducing insecticide efficacy by mediating efflux. Furthermore, ABC transporters are reportedly highly expressed in epidermal cells, where they may coordinate lipid transport and contribute to cuticle remodeling, thereby affecting both penetration and efflux of insecticides (Tarling, De Aguiar Vallim & Edwards, 2013; Chen et al., 2025).

Collectively, cuticular thickening and transmembrane efflux constitute a “physicochemical barrier” that serves as the first line of defense against insecticides. Although this form of resistance alone is usually insufficient to withstand lethal doses, it significantly enhances overall resistance when combined with target-site mutations and metabolic detoxification mechanisms.

### Metabolic resistance

Metabolic resistance is a systems-level reprogramming of xenobiotic handling that reduces the effective internal dose and accelerates clearance. The core enzyme families include cytochrome P450 monooxygenases, carboxyl- and cholinesterases, and glutathione S-transferases, with UDP-glycosyl/UDP-glucuronosyl transferases and ABC transporters contributing to Phase II and Phase III steps in some species. Common genetic bases involve promoter variation, copy-number amplification, and activation of upstream transcriptional programs, which together elevate expression or alter protein conformation and thereby generate cross-resistance across chemically diverse insecticides (Vontas, Katsavou & Mavridis, 2020; Zhou et al., 2022).

#### P450 monooxygenases

The P450 family detoxifies by oxidative inactivation or by generating substrates for Phase II conjugation and is a central route of resistance to pyrethroids and related insecticides in mosquitoes. Catalysis depends on NADPH–CYP reductase and is often enhanced by cytochrome b5. Field and laboratory studies repeatedly show high expression, copy-number amplification, or open-reading-frame mutations in specific P450s in resistant populations, with transcriptional control frequently linked to stress-responsive axes such as CncC/Keap1–Maf-S (Murataliev et al., 2008). Functional validation and multi-omics analyses in African anophelines consistently implicate CYP6M2 and members of the CYP6P subfamily (for example, CYP6P3) as metabolizers of multiple pyrethroids, and their transcriptional upregulation tracks with resistant phenotypes in heavily resistant Anopheles gambiae and Anopheles coluzzii populations. In Aedes, members of the CYP9J subfamily (such as CYP9J28 and CYP9J32) are closely associated with resistance, and CYP6Z8 catalyzes secondary oxidation of carboxylesterase-generated pyrethroid metabolites, converting 3-phenoxybenzyl alcohol and 3-phenoxybenzaldehyde into the less toxic and more readily excreted 3-phenoxybenzoic acid, illustrating a cooperative detoxification cascade with esterases (Smith et al., 2018). In the *Culex* pipiens complex, overexpression and amplification of CYP9M10 show a stable association with pyrethroid resistance and represent a lineage-specific yet mechanistically analogous solution. Evidence for direct P450-mediated detoxification integrates recombinant-enzyme metabolism, active-site mutagenesis, and piperonyl butoxide synergist bioassays (Poupardin et al., 2014). Tissue localization is enriched in midgut, fat body, and Malpighian tubules, consistent with a route of absorption, metabolism and excretion. Because individual P450s often display broad substrate spectra that promote multidrug cross-resistance, resistance management should combine synergists, rotation, and rational mixtures (Fotakis et al., 2022).

#### Esterases

Carboxyl/cholinesterases reduce effective contact between insecticides and their targets through hydrolysis or high-affinity sequestration. The canonical mechanism involves a first nucleophilic attack by the active-site serine on the carbonyl carbon of the ester bond to form an acyl-enzyme intermediate, followed by water-mediated deacylation that releases an acidic metabolite and regenerates the active enzyme (Paton et al., 2000). When esterase abundance is markedly elevated, reversible binding can further sequester toxicants. In *Culex*, amplification and overexpression at the Est*α*2/Est*β*2 locus are tightly associated with organophosphate resistance, with markedly increased total esterase activity that is stably inherited. In Aedes larvae, resistance to the organophosphate larvicide temephos is commonly linked to upregulation or amplification of CCEae3A and related esterases, and functional evidence supporting their causal role is accumulating (Sogorb & Vilanova, 2002). In adults, increased total esterase activity is also observed, although the specific driver genes can vary across geographies. For surveillance, synergist bioassays using S, S, S-tributyl phosphorotrithioate (DEF) or triphenyl phosphate (TPP) often partially restore susceptibility and provide a rapid field indicator. Integration with transcriptomic and copy-number analyses helps pinpoint candidate loci. Given the broad ester-bond specificity of many esterases, their upregulation can operate in tandem with P450 oxidation in a hydrolysis-first, oxidation-second cascade, reinforcing cross-resistance and improving clearance efficiency (Huang et al., 2023).

#### Glutathione S-transferases

GSTs are central to Phase II conjugation and to buffering oxidative stress, thereby playing dual roles in resistance. In insects, cytosolic GSTs predominate, with the Delta and Epsilon classes being most abundant. These enzymes catalyze conjugation of reduced glutathione to electrophilic insecticides or their metabolites to increase polarity and facilitate excretion (Gong et al., 2017). Some members also act as cofactors for dehydrochlorination during DDT detoxification and remove lipid peroxides and reactive oxygen species, limiting membrane injury and secondary toxicity induced by insecticides (Rault et al., 2019). In anophelines, a key amino-acid substitution in the Epsilon-class GSTE2 has been directly shown to confer high-level DDT resistance with collateral effects on pyrethroids, illustrating how structural variation and expression upregulation can combine to elevate detoxification capacity. In multiple field populations of Aedes, upregulation of Epsilon and Delta GSTs is common and aligns with pyrethroid resistance and mitigation of oxidative stress (Goindin et al., 2017). Microsomal GSTs are trimeric and may participate in membrane-associated metabolism, although their precise role in resistance remains incompletely defined. The *κ*-class GSTs are localized to mitochondria and peroxisomes in mammals and are absent from insects, so inferences from mammalian studies should be made with caution. For field inference, diethyl maleate synergist bioassays and enzyme activity assays can be combined with coexpression network analysis to assess whether GSTs act in parallel or in series with P450s and esterases, which helps identify metabolic bottlenecks and potential druggable targets (Zhong et al., 2013).

Although the resistance magnitude conferred by metabolic mechanisms is often moderate compared to target-site mutations, its cryptic nature makes it harder to detect through simple genotyping. Consequently, field monitoring typically relies on biochemical assays or gene expression profiling. In summary, reprogramming of detoxification networks represents a fundamental layer of resistance, enabling mosquitoes to reduce the bioavailability of insecticides through systemic metabolic adaptation.

Across mosquito taxa and ecological settings, metabolic resistance is seldom attributable to a single enzyme family acting in isolation. Instead, it reflects coordinated flux through multiple detoxification branches, including cytochrome P450 monooxygenase mediated oxidation, carboxyl and cholinesterase mediated hydrolysis or sequestration, and glutathione S transferase mediated conjugation and redox buffering. As a result, the relative contribution of each branch is best evaluated using quantitative readouts that can be compared across studies, including synergist bioassays that estimate pathway level causality, biochemical activity assays that approximate system capacity, and the magnitude of candidate gene induction or copy number amplification that expands detoxification throughput. In a southern Benin study using WHO diagnostic dose exposures, piperonyl butoxide pre-exposure produced marked and consistent increases in mortality to deltamethrin and permethrin, with absolute gains of approximately 61 and 39 percentage points, respectively, supporting a substantial but spatially variable role for P450 associated metabolism in field pyrethroid resistance phenotypes (Sagbohan et al., 2021). In the same study, biochemical assays indicated that carboxyl and cholinesterase activity and GST activity were elevated at some sites by roughly 1.6 to 1.9 fold relative to a susceptible reference, whereas bulk mixed function oxidase activity did not necessarily increase in parallel. This pattern is compatible with isoform specific or tissue restricted P450 overexpression that is not fully captured by coarse biochemical proxies, and it motivates integration of synergist assays with transcriptomic or targeted expression measurements to close the mechanistic loop. Evidence for parallel pathway engagement is also supported by synergist assays in Anopheles funestus from Nigeria, where diethyl maleate and S,S,S tributyl phosphorotrithioate pre exposures each restored permethrin mortality to 100 percent and increased DDT mortality to approximately 71 and 82 percent, respectively, indicating concurrent GST and carboxyl/cholinesterase contributions that can be quantitatively separated. Beyond pathway level inference, functional enzymology provides direct estimates of per enzyme detoxification efficiency and can reveal large effects of structural variation (Atoyebi et al., 2020). For example, the GSTe2 L119F variant in Anopheles funestus shows an approximately 3.4 fold higher catalytic efficiency toward DDT than the wild type enzyme and can metabolize a substantial fraction of permethrin *in vitro*, illustrating how amino acid substitutions can amplify detoxification flux and broaden effective substrate scope (Itokawa et al., 2010). Finally, the amplitude of capacity expansion differs markedly across taxa and enzyme systems, ranging from very high induction of carboxylesterase candidates such as CCEae3A in temephos resistant Aedes aegypti, to extreme overexpression of specific P450s such as CYP9M10 in pyrethroid resistant Culex quinquefasciatus, and to large copy number amplification at classical Culex esterase loci that increases transcript and protein abundance. Taken together, presenting synergist effects, biochemical activity shifts, candidate gene expression or copy number changes, and functional enzymology parameters side by side provides a comparable and transferable evidence framework and supplies a quantitative basis for translating metabolic resistance from descriptive mechanisms into field deployable biomarker panels (Table 1).

**Table 1:** Quantitative evidence for the relative contribution of major detoxification branches in mosquito metabolic resistance.

**Evidence type**	**Species and setting**	**Insecticide and class**	**Enzyme branch or marker**	**Reported quantitative metric**	**Approximate relative change**
Synergist bioassay (P450 associated)	Anopheles gambiae s.l., southern Benin	Deltamethrin and permethrin, pyrethroids	PBO sensitivity (proxy for P450 contribution)	Under diagnostic dose exposure, PBO increased mortality by about 61 and 39 percentage points (deltamethrin and permethrin, respectively)	Absolute mortality gain about 39 to 61 percentage points
Biochemical activity (carboxyl/cholinesterase and GST)	Same system, compared with susceptible reference	Same as above	Carboxyl/cholinesterase activity, GST activity, bulk MFO proxy	Carboxyl/cholinesterase and GST activities elevated at some sites, while bulk MFO proxy not consistently elevated	Roughly 1.6 to 1.9 fold increases for selected activities
Synergist bioassay (GST and carboxyl/cholinesterase associated)	Anopheles funestus s.s., Akaka Remo, Nigeria	Permethrin (pyrethroid) and DDT (organochlorine)	DEM (GST), DEF (carboxyl/cholinesterase associated)	Permethrin mortality restored to 100 percent with DEM or DEF; DDT mortality increased to about 71 percent (DEM) and 82 percent (DEF)	Restoration to complete kill for permethrin; partial restoration for DDT
Functional enzymology (GST variant efficiency)	Anopheles funestus, recombinant enzyme assays	DDT (organochlorine), permethrin (pyrethroid)	GSTe2 L119F versus wild type	Catalytic efficiency 316.3 versus 92.0 (mM^−^1 s^−^1) for DDT; substantial permethrin consumption in vitro	About 3.4 fold higher catalytic efficiency
Candidate gene induction (carboxylesterase capacity)	Aedes aegypti, field or selected populations	Temephos, organophosphate	CCEae3A and related carboxylesterases	CCEae3A reported as strongly induced in resistant populations	Greater than 60 fold induction
Candidate gene induction (P450 capacity)	Culex quinquefasciatus	Pyrethroids	CYP9M10	CYP9M10 reported as extremely overexpressed in resistant strains	About 260 fold overexpression
Candidate gene induction (P450 capacity)	Anopheles gambiae, resistant versus susceptible comparisons	Pyrethroid associated resistance	CYP6P3, CYP6M2	CYP6P3 about 12.4 fold; CYP6M2 about 7.4 fold	About 7 to 12 fold induction
Copy number amplification (esterase capacity)	Culex pipiens complex, classical amplified esterase loci	Organophosphates	Esterase amplification units	Copy number amplification with corresponding biased expression and increased protein abundance	Up to about 80 fold copy number expansion

### Microbiota and environmental adaptation

Endosymbiotic microorganisms and external environmental pressures have also been shown to modulate insecticide resistance in mosquitoes. The mosquito microbiome, particularly the gut microbiota, is considered an “invisible organ” that plays essential roles in host physiology, including detoxification and immune modulation (Wu et al., 2019b; Shi, Yu & Cheng, 2023).

Comparative studies have demonstrated that the composition of gut microbiota differs systematically between resistant and susceptible mosquito strains. Certain bacterial taxa are enriched in resistant strains and may contribute directly to resistance by metabolizing or inactivating insecticides. For instance, *Lactobacillus* species have been shown to secrete esterases that hydrolyze pyrethroids and organophosphates, thereby reducing their toxicity (Cui et al., 2024).

Experimental evidence supports this concept of microbial synergy: the introduction of exogenous bacteria such as *Streptococcus* or *Escherichia coli* into resistant *Anopheles* strains further increases resistance levels, whereas depletion of the gut microbiota using antibiotics renders previously resistant mosquitoes more susceptible (Mishra et al., 2022). These findings suggest that the microbiota may not only degrade insecticides directly but also modulate host gene expression. Some symbiotic bacteria have been shown to upregulate the expression of host P450 enzymes and antioxidant genes, effectively pre-conditioning the mosquito to resist insecticide exposure.

Beyond microbial contributions, environmental factors such as temperature and chemical stressors interact with genetic resistance to amplify insecticide tolerance. For example, *Aedes aegypti* mosquitoes exposed to fluctuating thermal environments exhibit increased tolerance to pyrethroids (Patel et al., 2024). The underlying mechanism may involve the upregulation of heat shock proteins and detoxification enzymes under thermal stress, which also enhances resistance to insecticides. This phenomenon is referred to as cross-tolerance or cross-protection, where a defense pathway activated by one stressor confers resistance to another (Yunta et al., 2016).

Environmental adaptation and microbiota changes often occur in tandem. In *Culex* mosquitoes inhabiting nutrient-rich polluted waters, both microbial community structure and host detoxification gene expression are altered, leading to a higher propensity for developing metabolic resistance (Reinhold et al., 2023).

Insecticide resistance in mosquito vectors is not determined solely by a single target-site mutation or an isolated metabolic pathway; rather, it represents a multilayered adaptive phenotype shaped jointly by host genomic background, transcriptional regulation and metabolic flux, symbiotic microbiota, and environmental pressures such as temperature variation and chemical pollution. Microbiota-mediated degradation of xenobiotics and induction of host detoxification programs, cross-tolerance triggered by non-insecticidal stressors, and the coordinated reshaping of microbial communities and host metabolic states in polluted habitats collectively indicate that resistance is characterized by network-level organization and strong context dependence. Consequently, reliance on a limited set of canonical genetic markers or single-omics indicators often fails to stably explain variation in resistance intensity and is insufficient to directly support key IVM decisions, including when to trigger insecticide rotation or mixtures, when to introduce synergists or biocontrol tools, and how to optimize intervention portfolios across distinct ecological and transmission settings.

A multi-omics framework is therefore necessary because it can integrate dispersed mechanistic signals into verifiable and transferable regulatory networks and biomarker panels, enabling resistance surveillance to shift from “single-point detection” toward “system-level early warning” and thereby establishing a translational chain from IR evidence to IVM action. Comparable paradigms have accumulated substantial experience in agricultural pests and insecticide resistance management: population genomics has been used to identify signatures of selection and structural variation, transcriptomics to delineate inducible detoxification responses, and proteomics and metabolomics to validate functional consequences in enzyme abundance and metabolic flux, collectively informing more targeted management recommendations. At the same time, this field has revealed shared bottlenecks, including limited transferability due to discrepancies between laboratory and field conditions, challenges in cross-platform and cross-batch comparability, sensitivity of network inference to annotation quality and parameterization, and the engineering difficulty of compressing high-dimensional omics signals into field-deployable diagnostic readouts. Building on these lessons and constraints, multi-omics studies in mosquitoes should be explicitly driven by IVM application scenarios, with emphasis on standardized cross-site and longitudinal surveillance designs, functional validation of key candidate mechanisms, and the construction of decision-oriented minimal biomarker panels to ensure that omics evidence can be converted into executable inputs for resistance management. On this basis, the next section will further discuss how multi-omics integration can be leveraged for regulatory network inference and to support actionable resistance early warning and optimization of IVM strategies.

## Integrative Multi-Omics for Regulatory Network Inference in Mosquito Insecticide Resistance

Integrative study designs that combine genomics, transcriptomics, proteomics, and metabolomics enable inference of resistance relevant regulatory networks and prioritization of biomarkers (Saavedra-Rodriguez et al., 2021). Cross-omics integration can consolidate layer-specific signals and transform candidate feature lists into testable network models, thereby improving mechanistic interpretability and supporting more transferable signatures across populations and settings.

### Genomics and population profiling

Genomic and population profiling aims to quantify how resistance-relevant variation is distributed across space, time, and taxa, and to infer the evolutionary processes that determine whether a candidate marker will be locally idiosyncratic or broadly predictive. The Phase 2 Ag1000G resource, comprising whole-genome variation data from 1,142 wild-caught *Anopheles gambiae* and *Anopheles coluzzii* sampled across 13 African countries, provides a continent-scale baseline for allele-frequency estimation and population-structure aware inference of resistance loci (The Anopheles gambiae 1000 Genomes Consortium, 2020). This breadth is essential because resistance alleles are embedded in heterogeneous demographic histories and admixture patterns that can mimic, amplify, or obscure selection signals when analyses ignore population context.

For target-site resistance, full-length analysis of the pyrethroid target gene *Vgsc* has revealed a more complex, multi-allelic architecture than the classic kdr narrative implies. In a whole-genome framework, Clarkson et al. (2021) resolved ten distinct kdr-associated haplotype groups and showed that several are present in multiple countries separated by more than 3,000 km, consistent with long-range spread of resistance haplotypes rather than purely local, de novo emergence. Beyond L995F/L995S, they identified 13 additional non-synonymous variants that occur almost exclusively on L995F-carrying backgrounds, supporting a model in which secondary substitutions can modify, compensate, or potentiate the core kdr effect. A particularly informative combination was the tight linkage between I1527T and V402L substitutions near a predicted pyrethroid-binding site, mirroring allele combinations implicated in resistance in other insect species and reinforcing the need to genotype beyond a single canonical codon.

Metabolic resistance frequently proceeds through structural variation that alters enzyme dosage, and genome-wide CNV discovery has made this mechanism directly measurable at scale. Using Ag1000G Phase 2 sequencing, Lucas et al. (2019) performed an agnostic CNV scan and reported that duplications are enriched in metabolic resistance gene families and often bear signatures of recent positive selection, indicating that copy-number increases can represent rapid adaptive responses to strong insecticide pressure. Within five metabolic “hotspot” regions, multiple independent duplications spanning the *Cyp6aa1* locus and neighboring genes showed selection-consistent patterns, highlighting gene amplification as a plausible route to elevated detoxification capacity even when no single coding substitution dominates. In contrast, fine-scale haplotype analysis of the metabolic gene *Cyp6m2* identified 15 high-frequency missense substitutions (>5% in at least one population) organized into five major haplotype clusters, yet found no evidence of directional selection acting on these coding variants or their haplotype backgrounds based on extended haplotype homozygosity decay. This discordance between frequent transcriptional implication of *Cyp6m2* and the absence of a coding-sweep signature supports the hypothesis that *Cyp6m2*-linked resistance may often be driven by distant regulatory factors or coordinated pathway shifts rather than by a single high-penetrance amino-acid change (Wagah et al., 2021).

Population genomics also clarifies how resistance emerges and disseminates across species boundaries under operational insecticide deployment. For organophosphate control, association mapping and replication across West Africa showed that pirimiphos-methyl resistance is best captured by a composite *Ace1* resistance haplotype combining copy number increase with a non-synonymous substitution, and that this haplotype evolved in *An. gambiae* before introgressing into *An. coluzzii* (Li et al., 2019). Extending locus-centric findings to genome-wide inference, a multi-country GWAS of resistance to deltamethrin and pirimiphos-methyl sequenced 969 phenotyped females and found that the genetic basis of resistance is highly multi-allelic and variable across populations, with the most consistent deltamethrin association centred on the *Cyp6aa1* region but mediated by multiple independent CNVs in *An. coluzzii versus* a non-CNV haplotype in *An. gambiae*.

The same GWAS also implicated shared regions such as *Cyp9k1* and *Tep* immunity genes across both insecticide classes, motivating surveillance designs that explicitly test for cross-class genetic architectures rather than assuming independence of mechanisms. To convert sparse, spatially biased genotyping into actionable decision support, Hancock et al. developed a Bayesian spatiotemporal model using 2,418 observations from 27 countries (2005–2017) and showed that ITN coverage is an influential predictor of *Vgsc* L995F/L995S frequency trajectories, with mapped allele frequencies providing a significant partial predictor of deltamethrin phenotypic resistance in *An. gambiae* complex populations (Yavaşoğlu & Şimşek, 2021).

Comparable genomic logic extends beyond *Anopheles*, illustrating how mapping designs can mechanistically anchor marker discovery in other vectors. In *Culex pipiens pallens*, QTL mapping linked a major pyrethroid-resistance locus to a clustered *CYP6* gene family, supporting the view that physically linked detox gene clusters can behave as selectable units and yield diagnostic markers when coupled to rigorous phenotype mapping. Promoter-level experiments in other insects further show that *CYP6* expression can be driven by inducible cis-regulatory architecture in response to xenobiotics, underscoring why noncoding variation and regulatory models deserve explicit consideration when “genomics” is used to explain metabolic resistance (Zou et al., 2019). Field evidence from *Culex pipiens* populations in Turkey reports heterogeneous combinations of target-site and enzymatic mechanisms across regions, reinforcing that genomic surveillance must be species- and geography-stratified to prevent overgeneralization of marker utility.

Genomics lays the foundational layer for understanding the heritability and evolution of resistance, while supplying key inputs for systems-level modeling and intervention strategies.

### Transcriptomics and the temporal dynamics of stress response

Transcriptomics provides an empirical bridge between genotypes and resistance phenotypes by quantifying how mosquitoes reprogram gene expression under both chronic selection and acute insecticide challenge. Beyond listing candidate detoxification genes, RNA sequencing enables mechanistic inference through study designs that explicitly compare resistant and susceptible backgrounds, profile inducible responses across time, and connect expression signatures to functional validation (Ingham et al., 2021). This evidence base has expanded across mosquito taxa and insecticide classes, showing that resistance is best interpreted as a dynamic, staged response rather than a static trait.

Comparative transcriptomic studies in field or selected laboratory populations repeatedly demonstrate that resistant mosquitoes exhibit coordinated upregulation of xenobiotic metabolism and cellular protection programs. In a deltamethrin resistant *Aedes albopictus* strain, genome wide RNA sequencing identified hundreds of differentially expressed genes, with enrichment of detoxification and stress response pathways; importantly, functional follow up supported causal roles for specific cytochrome P450 candidates (Wei et al., 2019). For example, CYP6A8 and CYP9B2 were implicated by differential expression and subsequently interrogated by RNA interference, illustrating how transcriptomic discovery can be closed into experimentally testable mechanisms rather than remaining descriptive (De Marco et al., 2017).

A key strength of transcriptomics is its ability to resolve temporal ordering after insecticide exposure, which is directly relevant to when and where surveillance should sample mosquitoes (Mappin et al., 2023). Time series profiling in *Aedes aegypti* following permethrin exposure, sampled at 6 h, 10 h, and 24 h, showed that transcriptional responses are sharply time dependent, with distinct sets of genes engaged at early *versus* later stages of recovery (Mack & Attardo, 2023). This design captures a progression from rapid induction of protective programs toward broader remodeling of metabolism, redox balance, and transport, emphasizing that candidate biomarkers and their detectability depend strongly on sampling time after exposure.

Similar temporal staging has also been demonstrated in Anopheles systems using controlled LD50 exposures. In Anopheles stephensi larvae challenged with a defined permethrin dose, RNA sequencing across 6 h, 24 h, and 48 h identified a substantial subset of “defensome” genes with time structured differential expression, supporting the concept that insecticide stress triggers a choreographed cascade rather than uniform upregulation of detoxification at all times (Azlan et al., 2019). Such datasets provide concrete benchmarks for separating immediate stress signaling from later phase detoxification and repair, and they motivate standardized time aware sampling frameworks for field transcriptomic surveillance.

Transcriptomics also substantiates that resistance relevant expression programs are not restricted to pyrethroids. For organophosphate selection, midgut RNA sequencing of a temephos resistant *Aedes aegypti* colony under long term selection showed broad transcriptional remodeling, including hundreds of upregulated genes spanning multiple detoxification enzyme families such as cytochrome P450s, glutathione S transferases, and glucosyl transferases, together with changes in cuticle associated processes and metabolic pathway enrichment (Mahmood et al., 2016). This provides primary evidence that metabolic resistance to organophosphates can involve extensive, tissue specific transcriptional shifts that would be missed by focusing on a small set of canonical pyrethroid markers.

In addition to detoxification enzymes, transcriptomic time series and perturbation studies have clarified the contribution of membrane transport and efflux programs, which can be invisible to classical target site genotyping. For example, *Anopheles gambiae* exposed to permethrin or bendiocarb exhibited insecticide induced upregulation of ABC transporter genes with clear temporal structure, and pharmacologic inhibition produced substantial synergism in bioassays, supporting an efflux component that is both inducible and operationally relevant (Mastrantonio et al., 2019). Integrating such expression signatures with bioassay-based synergy readouts strengthens causal interpretation and helps prioritize transporters as actionable biomarkers.

Mechanistic resolution improves further when transcriptomic signals are integrated with genomic variation. An illustrative population study in West African Anopheles gambiae combined expression profiling and genome wide association signals and identified a nonsynonymous variant in a sulfotransferase as strongly associated with resistance, while also showing that canonical kdr variation alone did not explain observed patterns in that dataset. This kind of integrated evidence supports the reviewer requested shift from single marker thinking toward network level signatures grounded in primary data.

Finally, transcriptomics has uncovered regulatory layers that can explain why detoxification genes are induced or constitutively elevated in resistant strains. MicroRNA mediated control provides experimentally tractable examples: in *Aedes albopictus*, miR-2b-3p showed an inverse relationship with deltamethrin resistance, and functional manipulation indicated that this microRNA can modulate resistance by repressing a P450 gene, linking noncoding regulation to detoxification capacity (Sun et al., 2019). Incorporating such regulatory modules into transcriptomic interpretation reduces overreliance on single gene lists and better reflects the causal architecture of resistance phenotypes.

Taken together, primary transcriptomic studies demonstrate that resistance associated expression programs are stage specific, tissue dependent, and class inclusive, spanning detoxification enzymes, transporters, redox control, and regulatory RNAs. For translation into resistance management, the most robust workflow is to define a small set of time stable and cross site reproducible transcriptomic markers, validate them with functional assays or synergy bioassays, and then embed them into routine surveillance as a complement to target site genotyping. This combination retains mechanistic interpretability while improving operational predictability under heterogeneous real world exposure histories.

### Proteomics: functional realization and post-translational modifications

Proteomics directly interrogates the effector layer of insecticide resistance by quantifying proteins that execute detoxification, barrier formation, transport, and stress adaptation. This layer is particularly informative because transcript abundance is not a reliable proxy for enzyme availability, complex assembly, or protein stability under selection. In pyrethroid resistant *Aedes aegypti* (CKR strain), a combined RNA seq and proteomics framework demonstrated that transcriptomic signals did not quantitatively mirror proteomic changes. Importantly, the study reported a set of CYPs showing concordant increases at both mRNA and protein levels, alongside additional CYPs elevated only at the protein level, supporting the idea that post transcriptional control or protein stabilization can contribute materially to resistance phenotypes (Sun et al., 2021).

Recent field focused quantitative proteomics has begun to provide experimentally anchored case studies that connect protein signatures to operationally relevant resistance traits and insecticide classes. In Penang Island *Ae. aegypti*, comparative proteomics of permethrin resistant adults and temephos resistant larvae identified substantial sets of differentially expressed proteins, with clear representation of structural and metabolic systems (Sookrung et al., 2018). For example, larvae comparisons identified a defined subset of proteins with significant differential expression between field resistant and laboratory exposed strains, providing a protein level basis for resistance associated physiology rather than purely conceptual pathway assertions (Zhang et al., 2021). In the same work, multiple cuticle associated proteins were highlighted among differentially expressed features, including pupal and larval cuticle proteins, supporting the view that barrier remodeling accompanies metabolic detoxification signals and can differ across life stages and exposure histories. Beyond structural proteins, the study also reported differential abundance of detoxification relevant CYPs such as CYP9J family members in resistant comparisons, illustrating that proteomics can recover enzyme level candidates that align with but are not fully captured by transcript only screening (Shettima et al., 2023).

Proteomic evidence for barrier based mechanisms is further strengthened by population and selection studies in other vector taxa. In *Culex pipiens pallens* subjected to insecticide selection, GO enrichment of upregulated proteins included structural constituent of cuticle, lipid transporter activity, chitin binding, and related functions, indicating that cuticle composition and lipid handling are repeatedly emphasized at the protein level under selection (Vanegas-Estévez et al., 2024). Consistently, mechanistic work on resistant malaria vectors has shown that reduced insecticide uptake can be associated with enhanced deposition of cuticular proteins and chitin together with increased cuticular hydrocarbons, reinforcing a proteomics supported, material barrier interpretation of penetration resistance (Balabanidou et al., 2019). These examples help shift the narrative from a general statement that “cuticle changes matter” to a reproducible protein signature that can be tracked and compared across studies and geographies (Sun et al., 2013).

Proteomics also expands the resistance map beyond detox enzymes by identifying systems that regulate protein turnover and stress resilience. A clear example is the identification of the proteasome subunit PSMB6 as a factor associated with deltamethrin resistance, where functional interference *via* knockdown reduced resistance in mosquito cells, providing causal support for a protein homeostasis axis in resistance biology (Zhang et al., 2022). This type of finding is difficult to infer confidently from genomics or transcriptomics alone and illustrates how proteomics can uncover non canonical contributors that may modulate the stability and effective activity of detoxification networks (Liu et al., 2021).

Although resistance focused datasets for post translational modifications remain less common than abundance profiling, the technical feasibility and biological informativeness of PTM aware proteomics are now well established in mosquitoes. For instance, phosphoproteomics in *Ae. aegypti* Malpighian tubules resolved thousands of phosphorylation sites across nearly two thousand phosphoproteins, demonstrating that pathway level signaling states can be quantified at scale (Kandel et al., 2022). Translating similar PTM aware designs to insecticide exposure and resistant *versus* susceptible contrasts would allow resistance models to incorporate kinase signaling, transporter regulation, and enzyme activation states, thereby improving mechanistic specificity and biomarker robustness.

Taken together, these primary studies support a more empirical framing of proteomics in mosquito insecticide resistance: proteomics validates whether candidate detox pathways are realized at the protein level, reveals parallel barrier and stress systems that co determine phenotype, and highlights regulatory layers such as protein turnover and PTM states that can decouple mRNA from functional capacity. Integrating these protein level readouts with transcriptomics and metabolomics can therefore move the omics sections from conceptual descriptions toward evidence anchored, testable biomarker candidates and intervention relevant pathways.

### Metabolomics: phenotypic signatures and energy–redox pathways

Metabolomics aims to comprehensively profile small-molecule metabolites in biological systems. Because metabolites lie at the downstream end of the molecular cascade linking genetic variation to cellular function, metabolomic readouts are often the closest omics layer to phenotype and can serve as a critical bridge between upstream regulatory events and observable traits (Rosli et al., 2024). In the context of mosquito insecticide resistance, metabolomics consistently indicates that resistant populations undergo metabolic reprogramming, with prominent signatures centered on enhanced energy supply and remodeled redox homeostasis. These phenotypic-endpoint data provide functional evidence for how mosquitoes accommodate sustained chemical stress and illuminate the physiological trade-offs accompanying resistance.

Current studies using nuclear magnetic resonance (NMR) spectroscopy and mass spectrometry platforms suggest that pyrethroid-resistant mosquitoes frequently display elevated tricarboxylic acid (TCA) cycle–related intermediates, including increases in metabolites such as pyruvate and citrate, together with reductions in products associated with amino-acid catabolism. Such patterns imply a redistribution of carbon flux toward mitochondrial energy metabolism, plausibly reflecting the increased energetic demand imposed by detoxification, transport, and damage-repair processes under insecticide exposure (Huang et al., 2021). Complementary liquid chromatography–mass spectrometry (LC–MS) profiling further shows that resistant strains can exhibit enrichment or strain-specific occurrence of diverse classes of metabolites, including organic acids, lipids, and carbohydrates. The implicated pathways span glycolysis, the TCA cycle, amino-acid metabolism, and phospholipid biosynthesis, supporting the view that resistance is not driven by a single pathway activation but rather by coordinated remodeling of interconnected metabolic modules (Vijay, Rawat & Sharma, 2014).

Beyond energy metabolism, alterations in redox balance and antioxidant capacity constitute another recurrent metabolomic theme in resistant mosquitoes. Increased abundance of glutathione-related metabolites and conjugated products such as sulfate conjugates has been reported in resistant backgrounds, consistent with reinforcement of antioxidant buffering and phase II conjugation reactions that mitigate insecticide-induced oxidative stress and accumulation of reactive toxic intermediates (Khemrattrakool et al., 2024). Mechanistically, these metabolite-level changes align with frequent observations from upstream omics layers showing induction of detoxification enzyme systems, and metabolomics provides a functional “downstream consequence” perspective that supports parallel engagement of detoxification and redox-protective programs in shaping resistance phenotypes (Li et al., 2022). Notably, signals related to lipid metabolism and membrane lipid remodeling are also commonly detected, which may influence membrane homeostasis, enzyme localization, and stress signaling, offering additional entry points for dissecting resistance-associated costs and adaptive benefits.

Overall, metabolomics reveals resistance-associated remodeling of energy-producing pathways and redox homeostasis at the phenotypic endpoint level and provides a principled basis for identifying metabolite signatures that reproducibly track resistance-related physiological states. When integrated with genomics, transcriptomics, and proteomics, metabolomics helps resolve which upstream genetic and regulatory events ultimately translate into measurable shifts in metabolic network activity, thereby strengthening mechanistic inference and moving resistance research from associative descriptions toward evidence chains that capture functional outcomes.

### Multi-omics integration: advancing from correlative insights to mechanistic networks

Systems-level integration approaches connect multi-layer evidence into coherent resistance networks and yield interpretable, transferable readouts. Network modeling, probabilistic inference, and machine learning frameworks provide principled routes to map multi-omic variation onto mechanistic hypotheses and to derive decision-ready outputs such as biomarker panels, pathway scores, and network-based risk indices.

By combining genomic, transcriptomic, proteomic, and metabolomic datasets, researchers can trace how variations at the DNA level influence gene expression, how these changes impact protein abundance and enzymatic activity, and ultimately how such modifications reshape cellular metabolism. This vertical integration enables the development of causal pathways linking genetic mutations to phenotypic resistance traits (Peinado et al., 2022).

Several recent studies highlight the value of such integrative approaches. For example, co-upregulated detoxification genes in resistant mosquitoes have been mapped onto the genome, revealing physical clustering that suggests regulation by shared transcription factors. These genomic regions often overlap with areas exhibiting signals of recent positive selection, as determined by population genetics analyses, providing strong evidence for their involvement in adaptive resistance (Amemiya, Horimoto & Fukui, 2021).

Parallel integration of proteomic and metabolomic data has facilitated the construction of resistance-related metabolic networks. By examining how enzyme-level alterations influence downstream metabolite concentrations, researchers have identified resistance-specific metabolic signatures and inferred upstream regulatory enzymes (Bouyssou et al., 2022). Such findings support the identification of biomarkers and contribute to the functional interpretation of omics data.

Probabilistic models, including Bayesian networks and dynamic inference systems, have been developed to quantify the likelihood and impact of specific resistance mechanisms. Meanwhile, machine learning algorithms, such as random forest classifiers, have successfully integrated environmental variables, allele frequencies, and gene expression data to predict the dynamics of resistance allele propagation under different intervention strategies (Bellone et al., 2023). These models enhance our ability to anticipate and manage resistance development using data-driven decision-making.

Multi-omics integration provides the analytical foundation to move beyond observational correlation and toward causal inference. By constructing interconnected resistance networks grounded in multi-level evidence, researchers are better equipped to elucidate existing resistance mechanisms, identify novel regulatory nodes, and forecast future resistance trajectories. This systems-level understanding is essential for guiding the design of more precise, proactive, and sustainable vector control strategies.

## Multi-omics-enabled Insecticide Resistance Management: from Surveillance to Spatiotemporal Deployment

**Figure 3:**
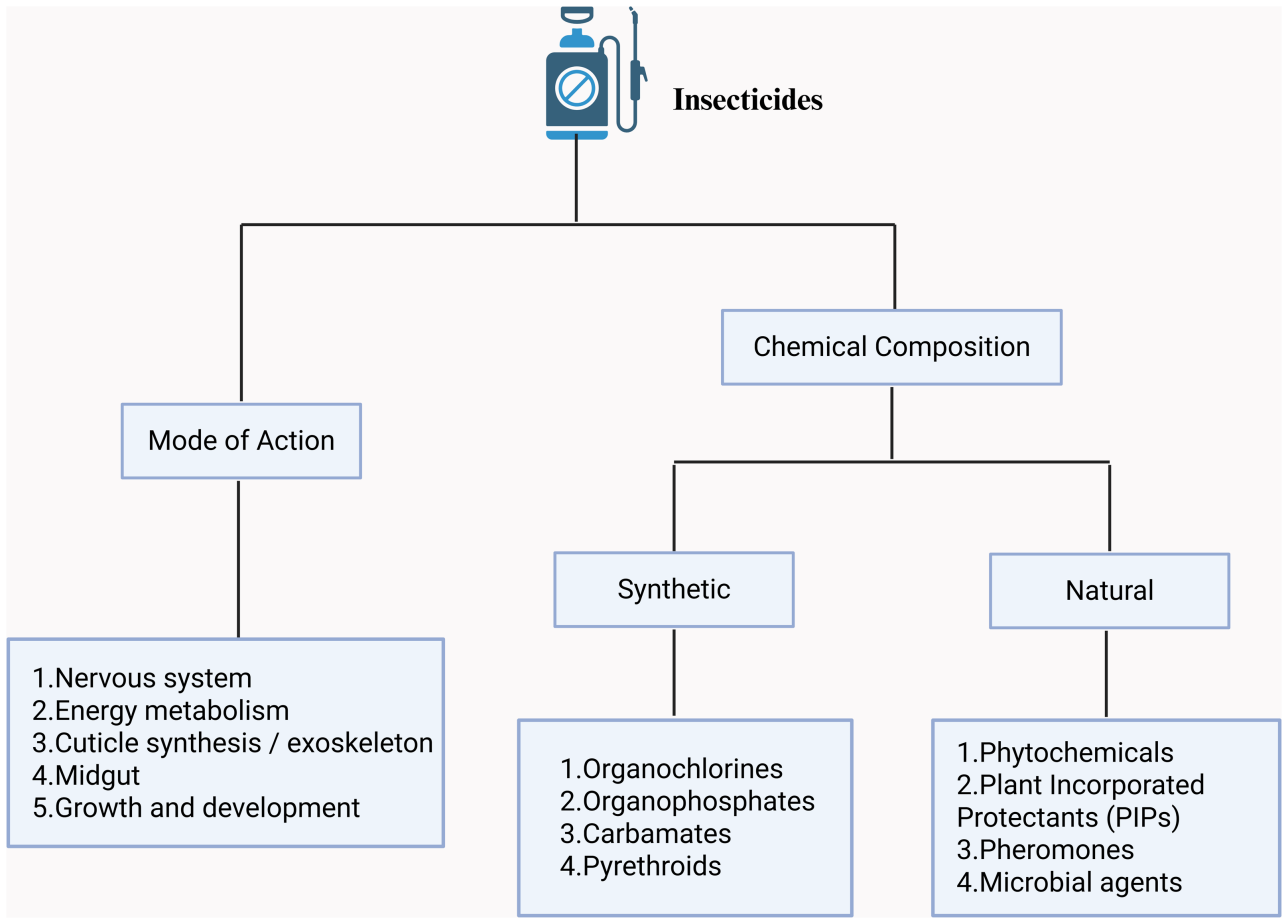
Classification of insecticides by mode of action and chemical composition. Insecticides organized along two axes. *Mode of action* includes effects on the (1) nervous system, (2) energy metabolism, (3) cuticle synthesis/exoskeleton, (4) midgut, and (5) growth and development. *Chemical composition* distinguishes synthetic (organochlorines, organophosphates, carbamates, pyrethroids) from natural (phytochemicals, plant-incorporated protectants, pheromones, microbial agents). The framework facilitates selection and management across categories. This figure was created by BioRender (https://www.biorender.com/) and does not involve any copyright issues.

Operationalization of omics-informed mechanisms and networks in insecticide resistance management requires standardized indicators, trigger thresholds, and explicit decision functions that translate surveillance outputs into insecticide choice and deployment (Tajudeen et al., 2023) (Fig. 3). Closed-loop frameworks that couple monitoring, modeling, and field outcomes allow iterative refinement of rotation, mixture, and spatiotemporal optimization strategies (Table 2).

### Indicator standardization and resistance risk stratification

The first step in precise IRM is the accurate measurement and quantification of resistance risk. Conventionally, resistance is diagnosed when mortality falls below a defined threshold, such as the WHO criterion of more than 10% survival after diagnostic dose exposure. Yet resistance is better understood as a gradient rather than a binary state. Recent approaches therefore advocate for multi-indicator stratification based on molecular, biochemical, and phenotypic markers.

Key indicators include allele frequencies of resistance-related mutations (such as kdr), quantified detoxification enzyme activities (including P450s, esterases, and GSTs), and refined phenotypic data such as dose–response curves or intensity scores (Wei et al., 2021). These variables can be normalized and integrated into a composite resistance index. For example, a polygenic resistance score (PRS) has been proposed to convert the effects of multiple resistance alleles into an equivalent predicted survival rate. A PRS value corresponding to 10% survival could serve as an operational threshold for intervention (Hobbs & Hastings, 2025b).

**Table 2:** Omics-Guided IRM overview: stratification, decision making, metabolomics, mixture strategies, and spatiotemporal precision.

Module	Objective	Key points (≤3)	Actions/Decisions
4.1 Indicators & Stratification	Quantify resistance risk on a continuous scale	Standardize multi-indicator panel (allele frequencies, enzyme activities, dose–response/intensity); PRS for polygenic resistance; calibrate with WHO diagnostic threshold (≥10% survival at diagnostic dose)	Define low/medium/high risk cutoffs; adopt a unified SOP for sampling, assays, and computation
4.2 Decision & Deployment	Select the optimal mix of rotation/mixtures/mosaic under local resistance and seasonality	Mixtures generally outperform single agents or rotation in polygenic resistance; real-time molecular diagnostics trigger mid-course adjustments; account for cross-resistance	If a resistance mechanism rises, drop the matching insecticide immediately; prefer low-overlap MoA pairs; set review intervals and trigger thresholds
4.3 Metabolomics for Rotation	Use metabolomics for early warning and sequence optimization	Track tryptophan pathway and GSH as early signals; assess A→B metabolic overlap; post-rotation biomarker reversion as success criterion	Pre-emptive rotation when metabolic signals rise; favor sequences with non-overlapping pathways; confirm success by marker decline toward baseline
4.4 Mixture Strategy	Delay resistance via dual-mechanism “two-hit” approach	Choose distinct MoA with low detox-pathway overlap; evaluate synergy and safety/cost	Prefer full-dose two-agent mixtures; avoid pairs metabolized by the same P450/detox enzyme
4.5 Spatiotemporal Precision	Tailor deployment by space and season	Spatial mosaic zoning; seasonal intensity tuning; GIS resistance maps for targeted interventions	In high-resistance zones, replace agents selectively while maintaining neighbors; use mixtures/rotation at peaks; downscale in low season

Some studies have developed threshold-based classifications that combine enzyme activity levels, mutation frequencies, and mortality assays to categorize mosquito populations into low-, moderate-, or high-risk tiers. This stratification is particularly valuable for prioritizing interventions under resource-limited conditions (Devillers et al., 2023).

Standardization of measurement protocols is essential to ensure comparability across studies. Both WHO and national CDCs have updated resistance monitoring guidelines to include molecular diagnostics and enzyme inhibition assays that help identify resistance mechanisms. As new omics-based markers become available, such as metabolite indicators, they may be incorporated into field-ready assays for early-warning applications (Sparks et al., 2021).

A unified resistance assessment framework based on multiple standardized indicators is the foundation of omics-informed IRM. It provides a consistent language for interpreting resistance risk and facilitates data-driven decision-making.

### Unified decision functions and insecticide deployment strategies

Once standardized indicators are available, the next challenge is to establish decision rules for selecting and adjusting interventions. Core IRM strategies include temporal rotation, chemical mixtures, and spatial mosaics. However, determining the optimal approach for a specific region requires consideration of local resistance profiles and seasonal transmission dynamics.

Mathematical models can assist in evaluating the long-term effects of different IRM strategies on resistance allele frequencies. For example, one recent simulation study showed that although rotation can reduce peak resistance levels, it does not substantially extend the overall effective lifespan of insecticides compared to continuous application (Sisterson, 2022). In contrast, full-dose mixtures of two insecticides with different modes of action significantly delayed resistance progression and provided the longest protective duration (Killeen, 2020). These findings suggest that combination strategies may be more effective than rotation, especially in polygenic resistance scenarios.

Real-time molecular diagnostics can further refine intervention timing and choice. For example, if field assays detect elevated levels of resistance mutations or increased expression of detoxification enzymes, immediate adjustments can be made to exclude the corresponding insecticides. Some portable devices can identify key mutations such as kdr within 30 min, enabling mid-season intervention rather than waiting for end-of-season reports.

Effective decision systems must also account for cross-resistance. If metabolic pathways confer resistance to both a current and candidate insecticide, the latter should be avoided or delayed in deployment. Ideally, these considerations are integrated into an algorithmic decision-support platform. The system would accept input on resistance indicators, vector species composition, and mosquito density, and then output optimal recommendations such as maintaining, switching, or combining insecticides. It would also suggest the timing of follow-up assessments.

Although algorithm-driven decision tools improve consistency and responsiveness, expert interpretation and adaptation to local realities remain essential. Overall, omics-informed IRM strategies are expected to be more sensitive, flexible, and rational, thereby maximizing the longevity of insecticides and sustaining vector control outcomes.

### The role of metabolomics in insecticide rotation strategies

Metabolomics holds particular promise for insecticide resistance management, as metabolic alterations often precede overt phenotypic resistance, making them ideal tools for early warning and risk assessment. One envisioned application is periodic collection of mosquito samples for metabolic fingerprinting. If metabolomic signals associated with a known resistance mechanism begin to intensify, it may indicate the early development of resistance, prompting preemptive rotation of the corresponding insecticide class (Singh et al., 2022). This proactive approach improves upon traditional strategies that wait for failure in bioassays before modifying interventions.

Recent studies have proposed several candidate metabolic biomarkers linked to resistance. For example, metabolomic profiling of pyrethroid-resistant *Aedes albopictus* revealed significant changes in tryptophan metabolism. It was hypothesized that enhanced tryptophan degradation may help eliminate neurotoxic byproducts induced by pyrethroid exposure (Madgwick & Kanitz, 2024). If confirmed, tryptophan metabolites could serve as indicators for resistance monitoring. Similarly, elevated glutathione levels, which are associated with oxidative detoxification, may act as broad-spectrum markers of emerging resistance. A detectable rise in glutathione or its derivatives in field mosquito populations could signal incipient resistance, even in the absence of known resistance mutations.

Metabolomics also aids in optimizing the sequence of insecticide rotations. Different insecticides induce distinct metabolic responses. Analyzing these patterns reveals which physiological pathways are mobilized following exposure to one chemical, and whether these adaptations influence susceptibility to subsequent agents (Malla et al., 2023). For example, if treatment with compound A upregulates enzymes that also metabolize compound B, then using B immediately after A may hasten resistance to B and should be avoided. Conversely, if the metabolic pathways activated by one insecticide are non-overlapping or even reversed upon switching to another, then the rotation is likely to be effective (Stuart et al., 2023). This insight goes beyond traditional views of cross-resistance by considering the time-dependent physiological plasticity of insects.

Furthermore, metabolomic profiling can be used to evaluate the effectiveness of a rotation strategy. After a period of alternating insecticides, researchers can assess whether the metabolic markers associated with resistance to a previously used compound have returned to baseline. A decline in resistance-associated metabolites suggests that the population has regained susceptibility, validating the success of the strategy (Elzaki et al., 2020). If not, adjustments to the rotation interval or drug combination may be necessary.

Metabolomics enhances IRM by offering a forward-looking and precise framework. It enables early detection of resistance trends, informs the timing of rotations, facilitates the selection of complementary compounds, and supports post-rotation evaluation. This leads to scientifically grounded and adaptable resistance management strategies tailored to real-world complexities.

### Mixture strategies: balancing synergy and cross-resistance

Beyond rotation, the use of insecticide mixtures is another core IRM strategy, involving the simultaneous or combined application of multiple compounds with distinct modes of action. The theoretical advantage is that, provided there is no cross-resistance, mosquitoes must acquire two independent resistance mechanisms to survive, substantially slowing the pace of resistance development (Hobbs & Hastings, 2025a). Modeling studies have confirmed this benefit, showing that in polygenic resistance scenarios, full-dose mixtures significantly extend insecticide lifespan compared to sequences or rotations, even when partial cross-resistance is present.

However, successful mixture strategies must be carefully designed to avoid unintended consequences. Ideally, the combined agents should exhibit synergistic effects, such as enhanced penetration or increased lethality, enabling lower doses of each to be used while maintaining efficacy (Scharf & Lee, 2024). At the same time, if both compounds are metabolized *via* the same detoxification pathway, the mixture may intensify selection pressure on that mechanism, accelerating the emergence of multi-resistance.

The Insecticide Resistance Action Committee recommends that mixtures consist of components with unrelated modes of action and no shared resistance mechanisms. In mosquito control, practical options include co-spraying spatial interventions with two agents, treating bed nets with dual insecticides (such as a pyrethroid plus a novel non-pyrethroid), or combining larvicides with adulticides in integrated management (Pu & Chung, 2024).

Several successful case studies support this approach. For instance, co-formulating a new diamide compound with a low-dose pyrethroid has been shown to restore efficacy against pyrethroid-resistant mosquito populations, extending the utility of bed nets (Burns & Pastoor, 2018). Nevertheless, mixtures often incur higher costs and raise concerns about safety for non-target organisms and human populations.

Omics data can help refine mixture decisions. Metabolomic and transcriptomic profiles can reveal whether overexpressed resistance enzymes affect both candidate compounds. If a single cytochrome P450 enzyme metabolizes both agent A and agent B, they should not be mixed. Conversely, if resistance to agent A is mediated by a target-site mutation and resistance to agent B involves metabolic detoxification, their mixture poses a dual challenge for the mosquito and is more likely to be effective.

In essence, the success of a mixture strategy hinges on achieving synergy while avoiding overlap in resistance pathways. Rational mixture design should be guided by deep understanding of both pharmacological and resistance mechanisms and supported by empirical field data. When carefully implemented, mixtures offer a powerful tool for delaying resistance, particularly in areas with already high resistance prevalence.

### Spatiotemporal precision and resource optimization

Mosquito insecticide resistance is not uniformly distributed; instead, it exhibits pronounced spatial and temporal heterogeneity. Variation in vector species composition, ecological gradients, and seasonally patterned insecticide use produces locally distinct selection pressures, resulting in resistance landscapes that shift across both geography and time. Effective IRM therefore benefits from strategies that are explicitly localized and time-aware, enabling adaptive deployment that maintains intervention effectiveness while minimizing unnecessary selection pressure and optimizing limited operational resources.

Along the spatial axis, mosaic deployment partitions control areas into operational zones that receive different insecticide classes, thereby reducing uniform selection pressure and preserving susceptibility at broader landscape scales (Owusu-Asenso et al., 2022). When surveillance indicates elevated resistance in one zone but not in adjacent areas, selective rotation, substitution, or augmentation can be implemented where needed rather than applying synchronized changes across an entire region, maximizing the utility and cost-effectiveness of existing compounds (Kim et al., 2020). In highly heterogeneous urban settings, GIS-informed resistance maps can support fine-grained targeting, prioritizing interventions in hotspots where marginal returns are highest while avoiding overtreatment in lower-risk areas (Dangbenon et al., 2025). Because mosquito movement and human mobility can facilitate the spread of resistance, coordinated cross-border or cross-jurisdictional monitoring and response are also essential to prevent localized hotspots from undermining wider regional control efforts.

Temporally, intervention intensity and insecticide schedules should be aligned with seasonal vector dynamics. During periods of lower vector abundance or reduced transmission risk, chemical pressure can be attenuated to limit redundant selection and slow resistance escalation. During peak seasons, more intensive measures, including rotations, mixtures, or staged combinations, may be warranted to sustain efficacy. In some contexts, a phased approach can be operationally advantageous, emphasizing biological or environmental larval control during larval peaks, strengthening adult control during adult peaks, and switching to an alternative mode of action in the post-peak phase to avoid prolonged dominance of a single selection regime (Wilcox et al., 2021). The practical value of such temporal stratification lies in converting seasonal windows into actionable scheduling rules that simultaneously track transmission risk and constrain cumulative selection pressure.

Omics and integrated data streams can further refine spatiotemporal management, but their contribution should be expressed as decision-ready inputs rather than reiterative conceptual claims. Comparative genotyping and population genetic analyses across regions can trace the origin and movement of resistance alleles, informing earlier intervention at sources and along dissemination corridors. Seasonal monitoring of molecular and physiological indicators can complement routine surveillance by highlighting periods of elevated resistance risk and supporting more precise timing of interventions. When combined with sentinel surveillance networks, environmental and meteorological covariates, and spatial analytical models, these data can improve prediction of hotspot emergence and evolution, enabling risk-informed allocation of resources and timely updates to deployment strategies.

Spatiotemporal precision emphasizes differentiated and adaptive answers to where to deploy which tools, and when and how intensively to deploy them. By translating surveillance outputs into clear trigger thresholds and predefined response options, IRM can move toward an optimized resource-allocation paradigm that balances immediate effectiveness with long-term sustainability (Fig. 3).

## Operationalizing omics-enabled IRM: closing the loop from surveillance to adaptive deployment

Building on the multi-omics-enabled IRM framework, this section turns to operationalization, constructing a surveillance-to-deployment-to-refinement loop along three pillars: rapid diagnostics, city-level pilots, and integrated chemical and nonchemical interventions. The overarching objective is to establish a pathway from data to decisions in real-world settings and to drive continuous optimization through feedback, allowing strategies to adapt to local resistance ecologies and seasonal dynamics.

### Field-deployable molecular and metabolic diagnostics with actionable thresholds

For frontline vector control teams, access to rapid and simple diagnostic tools is essential to support timely decision-making. The development of field-applicable technologies for resistance monitoring is therefore a priority. In the molecular domain, traditional PCR and sequencing require laboratory infrastructure, but portable diagnostic platforms are closing this gap. For example, researchers in the United Kingdom have developed a portable PCR chip for detecting *kdr* mutations in field-collected mosquitoes. With just a small device and paper-based cartridge, this system can detect L1014F/S and N1575Y mutations within an hour (Tepa et al., 2022). Its results are fully consistent with laboratory-based TaqMan assays, achieving 100% sensitivity and specificity.

Isothermal amplification methods, such as loop-mediated isothermal amplification (LAMP), have also been applied to detect *Ace-1* mutations without the need for sophisticated equipment, making them particularly suitable for low-resource settings (Sharma et al., 2022). Rapid detection of gene expression has also been explored; for instance, reverse-transcription LAMP can be used to identify overexpression of key cytochrome P450 genes, serving as a proxy for metabolic resistance (McGinnis et al., 2021).

On the biochemical side, enzymatic activity can be quickly assessed using reagent strips or biosensors. Traditional enzyme activity assays on paper strips have been used to estimate esterase or oxidase levels semi-quantitatively. More advanced approaches involve portable Raman or near-infrared (NIR) spectrometers that scan intact mosquitoes (Pang et al., 2020). These devices detect differences in chemical composition that reflect resistance-related metabolic changes. Studies have shown that NIR spectroscopy can distinguish resistant from susceptible individuals in seconds, with reported accuracy ranging from 80 to 90 percent under certain conditions (Li et al., 2021).

However, rapid detection methods are only useful when paired with well-defined thresholds for intervention. That is, predetermined action thresholds must be established to translate results into operational decisions. For example, if the frequency of *kdr* alleles surpasses 50 percent, an insecticide class switch may be triggered. Similarly, if enzymatic activity exceeds a twofold increase relative to susceptible controls, synergist bioassays may be warranted. With clear decision thresholds, frontline workers can act immediately based on field results (Wu et al., 2023).

To enhance scalability and responsiveness, a digital platform should be established to enable real-time data entry and sharing. Field diagnostic results should feed directly into centralized databases, allowing broader-level monitoring and adaptive strategy planning. Looking ahead, CRISPR-based diagnostics and mobile phone-compatible readers may further simplify resistance testing. These innovations could enable farmers or field workers to detect insecticide resistance with the ease of water-quality testing, dramatically expanding the scope and frequency of surveillance. This would make IRM more data-driven and operationally responsive.

### Pilot-to-scale implementation of a surveillance–modeling–decision feedback loop

Before scaling up omics-informed IRM approaches, it is crucial to validate the full-loop system through pilot programs. Selecting high-priority regions (such as cities with high mosquito-borne disease burdens and well-documented resistance issues) is a strategic starting point. These pilots can serve as controlled environments for establishing and testing integrated workflows from surveillance to decision-making.

In such a pilot program, intensive resistance monitoring should be implemented, including routine phenotypic bioassays, molecular marker screening, and targeted omics profiling of priority genes and metabolic biomarkers. All data collected should be entered into a centralized resistance database in real time (Wu et al., 2024).

Simultaneously, modeling teams should be engaged to simulate and forecast the impact of various IRM strategies based on local vector species composition, seasonal patterns, and past intervention records. Using evolutionary models and machine learning algorithms, these teams can generate dynamic predictions. As new surveillance data are fed into the system, the models can update resistance trajectory forecasts and recommend optimal intervention options.

These recommendations are communicated *via* a decision-support platform to public health authorities. Experts then translate model outputs into practical action plans, such as when to rotate insecticides, whether to deploy combination tools, or whether to intensify interventions. Once implemented, further field monitoring is conducted to assess whether resistance trends have stabilized or reversed. If so, the strategy is validated. If resistance continues to rise, model parameters and strategies are adjusted accordingly, and a new iteration of the loop begins.

Such a system constitutes a continuous monitoring–modeling–decision–feedback loop, steadily converging toward optimal solutions. During the pilot phase, particular attention should be given to evaluating cost-effectiveness and operational feasibility. For example, how frequently and how extensively must monitoring be conducted to enable accurate predictions? Will data latency or uncertainty compromise decision quality? If strategies require frequent updates, can this be accommodated within existing vector control cycles?

Small-scale pilots offer opportunities to troubleshoot these questions. Some omics indicators may prove impractical under field conditions, or environmental covariates may need to be added to improve model performance. The pilot also facilitates institutional learning by identifying which forms of collaboration—such as between health departments and academic researchers—are most effective. Moreover, pilot programs can be used to train a new generation of interdisciplinary professionals who understand both molecular diagnostics and computational modeling, ensuring long-term implementation capacity.

Ultimately, successful pilots can be scaled up and replicated. In a broader sense, this feedback loop exemplifies a form of adaptive governance. Like an autoregulation system, IRM strategies evolve through cycles of trial and correction. If the system proves effective, it will not only curb resistance locally but also serve as a model for data-driven precision control efforts globally.

### Integrating chemical and nonchemical interventions for durable control

The ultimate goal of precision IRM is to maintain disease control efficacy while minimizing reliance on chemical insecticides. To achieve this, IRM must be integrated with non-chemical vector control measures, forming a comprehensive and sustainable solution. Currently, the most promising non-chemical approaches include the use of *Wolbachia* symbionts, environmental habitat management, and genetic control strategies (Shemshadian et al., 2021).

*Wolbachia* are maternally inherited endosymbiotic bacteria, and certain strains have been shown to reduce the ability of mosquitoes to transmit arboviruses. Field trials deploying *Wolbachia*-infected mosquitoes have already been conducted in several cities to combat dengue outbreaks (Tantowijoyo et al., 2022). When combined with insecticide-based strategies, the deployment of *Wolbachia* offers dual benefits. First, the reduced vector competence decreases the urgency for insecticide use, since surviving mosquitoes are less likely to transmit pathogens. Second, the reduced insecticide pressure helps delay the development of resistance, thereby enhancing the durability of *Wolbachia*-based interventions (Garcia et al., 2020). Experimental data even suggest that the simultaneous release of *Wolbachia*-infected mosquitoes and limited insecticide application can synergistically suppress wild mosquito populations more rapidly (Ateutchia-Ngouanet et al., 2024).

Environmental habitat management, such as removing water-holding containers and improving drainage systems, reduces mosquito breeding sites at the source. This strategy is particularly effective in alleviating adult mosquito control pressure in regions with high levels of resistance, thereby providing a respite for insecticides. In addition, genetic control methods include the sterile insect technique (SIT), genetically engineered sterility using lethal gene constructs, and gene drive systems (Pérez-Staples, Díaz-Fleischer & Montoya, 2021). These technologies directly reduce mosquito population density without the use of chemical agents and thus serve as alternatives or supplements to conventional insecticides.

Nevertheless, these innovative technologies also present limitations. For example, SIT requires sustained releases and is associated with high operational costs. Gene drive approaches raise ecological and ethical concerns (Dumont & Yatat-Djeumen, 2022). Therefore, integrative strategies should capitalize on the strengths of each method while compensating for their weaknesses. For instance, *Wolbachia* releases may be prioritized in densely populated urban areas, while larval habitat management can be emphasized in peri-urban or rural regions. During peak transmission seasons, sterile male mosquito releases may be combined with conventional spraying campaigns to further reduce population density.

Such multi-pronged interventions introduce complex selection environments that distribute adaptive pressures across different control modalities. Even if resistance to insecticides arises, the overall vectorial capacity of the mosquito population may be diminished due to infertility or reduced pathogen competence, thereby weakening the adverse impact of resistance (Maia et al., 2023). This aligns closely with the philosophy of precision control, where locally tailored strategies are designed to achieve maximal efficacy while minimizing unintended consequences such as resistance proliferation or environmental contamination.

Omics data can also enhance the implementation of non-chemical strategies. For example, population-level genotyping can monitor the introgression of *Wolbachia* into wild mosquito populations and guide decisions regarding supplementary releases. Likewise, genetic structure analyses of mosquito populations can inform the scale and frequency of sterile male releases to ensure sufficient coverage of the target gene pool (Fifer et al., 2025). Furthermore, microbiome studies may identify other symbiotic microorganisms with potential to impair mosquito fitness or pathogen transmission, serving as novel candidates for *Wolbachia*-like interventions.

The major challenge of integrated strategies lies in coordinating among multiple sectors and ensuring long-term resource commitment. However, the advantage is the creation of a diversified intervention landscape that does not rely exclusively on any single method. This makes it more difficult for mosquitoes to adapt simultaneously to all forms of control. Ultimately, the integration of chemical and non-chemical approaches represents the future of vector management and the most promising solution to overcome the challenge of resistance. Only by constructing an ecologically informed, multifaceted control framework can sustainable management of mosquito-borne diseases be achieved. We summarized the clinical Trials Related to Mosquito-Borne Insecticide Resistance (Table 3).

**Table 3:** Clinical trials related to mosquito-borne insecticide resistance.

NCT number	Study title	Conditions	Interventions	Phases
NCT01160809	Effect of a Combined Use of Mosquito Repellent and Insecticide Treated Net on Malaria in Ethiopia	Malaria—Mosquito Repellent—Long Lasting Insecticidal Nets	OTHER: Mosquito repellent	PHASE4
NCT01713517	Impact of Insecticide Resistance on Vector Control	Malaria, Falciparum	OTHER: Indoor residual insecticide spraying (IRS)—DEVICE: Long Lasting Insecticidal Nets (LLIN)	PHASE4
NCT06268691	Sustainable Reduction of Dengue in Colombia: Vector Breeding Site Intervention With an Insecticidal Coating	Dengue—Vector Borne Diseases—Arbovirus Infections—Zika—Chikungunya Fever	OTHER: Insecticide Coating INESFLY	NA
NCT02458066	Trial to Compare Effectiveness of 2 Insecticides in Preventing Malaria	Malaria	OTHER: IRS: bendiocarb—OTHER: IRS: deltamethrin	NA
NCT00169078	Impact of Insecticide-treated Curtains on Antimalarial Drug Resistance	Malaria	DRUG: Chloroquine	NA
NCT02938975	Field Efficacy Of Insecticide Treated Uniforms And Skin Repellents for Malaria Prevention	Malaria	OTHER: Ultra 30 Insect Repellent Lotion (30% Lipo DEET)—OTHER: Permethrin Factory-Treated Army Combat Uniforms—OTHER: Placebo lotion—OTHER: Army combat uniform	PHASE3
NCT02448745	Liberia Insecticide Treated Durable Wall Linings Study: Protocol for a Cluster Randomised Trial (DL)	Malaria, Falciparum—Anemia	DEVICE: Insecticide treated wall lining	NA
NCT02533336	The Effectiveness of Non-Pyrethroid Insecticide-Treated Durable Wall Liners as a Method for Malaria Control in Endemic Rural Tanzania	Malaria	DRUG: abamectin and fenpyroximate	PHASE3

## Challenges and Future Perspectives

Despite the transformative potential of multi-omics technologies for dissecting and forecasting insecticide resistance in mosquito vectors, several barriers still limit translation from mechanistic insight to operational IVM. A central challenge is that omics signals are often generated under heterogeneous sampling designs, phenotyping protocols, and ecological contexts, making cross-study comparability and actionability difficult. Resistance phenotypes are typically inferred from bioassays performed under different exposure regimes, life stages, and insecticide formulations, while omics readouts are influenced by age, blood-feeding status, microbiome composition, and local environmental stressors. Without harmonized metadata, standardized phenotyping, and longitudinal sampling across sentinel sites, it remains challenging to distinguish causal resistance mechanisms from context-dependent correlates, and to define robust biomarkers that generalize across settings.

A second challenge concerns the interpretation of multi-layer data into decision-grade evidence. Omics datasets can be noisy and high dimensional, and network inferences or machine-learning predictions may be sensitive to parameter choices, batch effects, and incomplete annotations of mosquito genomes and gene families. Importantly, the operational question in IVM is rarely whether resistance exists, but whether its magnitude and trajectory warrant changing interventions. This requires calibrated links between molecular signatures and quantitative outcomes such as resistance intensity, mortality under realistic exposure, and intervention effectiveness. Key knowledge gaps include incomplete genotype-to-phenotype mapping across target-site mutations, copy-number variation and structural variants, regulatory changes driving metabolic resistance, and epistatic interactions that shape cross-resistance. Multi-omics integration should therefore prioritize causal and experimentally anchored frameworks, including functional validation of candidate genes and pathways, orthogonal confirmation of expression at the protein level, and metabolite-based readouts of detoxification flux, rather than relying solely on statistical associations.

Third, operationalization depends on converting complex omics outputs into field-deployable assays and actionable thresholds. While whole-genome sequencing, transcriptomics, proteomics, and metabolomics can identify resistance mechanisms and predictive signatures, routine surveillance programs require low-cost, rapid, and interpretable diagnostics. A practical roadmap is to use discovery omics to nominate a minimal panel of validated markers that can be monitored with targeted sequencing or rapid molecular tests, coupled with predefined decision thresholds that trigger insecticide rotation, mixture deployment, or the introduction of synergists. Establishing these thresholds is itself a major requisite: it demands prospective studies that jointly track molecular markers, bioassay-derived resistance intensity, and programmatic outcomes under real intervention conditions. Without such prospective validation, “precision resistance management” risks remaining conceptual.

Beyond technical considerations, socioeconomic and governance constraints are often the decisive determinants of adoption. High-throughput surveillance entails costs for consumables, instrumentation, cold chain, data storage, and skilled personnel, and these costs are concentrated in precisely the regions where vector-borne diseases are most endemic. Supply-chain fragility, procurement cycles for long-lasting insecticidal nets and indoor residual spraying, and the limited flexibility of budgets can delay timely switching of interventions even when molecular evidence indicates rising resistance risk. Community acceptance and regulatory pathways also shape feasibility, particularly for biocontrol and genetic strategies. Data governance, including data sovereignty, equitable benefit sharing, and transparent communication of uncertainty, is essential to sustain trust among national programs and communities. Consequently, implementation research should incorporate health-economic evaluation, including cost-effectiveness under realistic constraints, workforce training needs, and institutional coordination mechanisms linking public health agencies, research laboratories, and local stakeholders.

Looking forward, multi-omics can play a decisive role in guiding both molecular and biocontrol advances within an expanded IVM toolbox. On the molecular side, next-generation insecticides targeting alternative pathways, mixtures and rotations, and synergist-based approaches can be informed by multi-omics profiling of target-site variation, detoxification networks, and metabolic pathway rewiring that underlies cross-resistance. Proteogenomic and metabolomic evidence can help distinguish transcriptional induction from functional enzyme abundance and detoxification capacity, improving the prediction of which chemistries are likely to fail or remain effective. Multi-omics can also support proactive resistance risk assessment for newly deployed tools by identifying early adaptive signals before phenotypic resistance becomes operationally evident.

On the biocontrol front, interventions such as Wolbachia-based population replacement, larval biolarvicides (*e.g.*, Bacillus thuringiensis israelensis), entomopathogenic fungi, and habitat-based strategies may exhibit variable performance across ecological settings. Integrating host genomics, microbiome profiling, immune and metabolic readouts, and environmental covariates offers a path to mechanistically explain heterogeneity in field outcomes and to optimize deployment strategies. For genetic control tools, including gene-drive-based approaches, multi-omics surveillance is indispensable for monitoring population structure, introgression, potential resistance alleles at drive targets, and unintended ecological effects, while governance frameworks must address ethical, regulatory, and societal considerations in parallel.

In summary, the field now needs a translational bridge that connects IR discovery to IVM action: harmonized sampling and phenotyping, validated multi-omics biomarkers with causal support, simplified diagnostics suitable for routine surveillance, and decision frameworks with explicit thresholds and cost-informed feasibility. Addressing these requisites through coordinated, longitudinal, and policy-integrated research will enable multi-omics to move from descriptive insight to operational impact, improving the sustainability and effectiveness of mosquito control programs in the face of rapidly evolving resistance.

## Conclusion

Insecticide resistance in mosquito vectors remains a primary constraint on the durability of vector-borne disease control. Evidence consistently supports a multilayered adaptive process spanning molecular, cellular, organismal, and ecological scales. Target-site substitutions reduce receptor and channel sensitivity, metabolic reprogramming through cytochrome P450s, glutathione S-transferases, and carboxylesterases broadens detoxification capacity, and physical–chemical defenses such as cuticular thickening and transmembrane efflux limit internal dose. Microbiome composition and environmental stressors further modulate these phenotypes and shape evolutionary trajectories.

The maturation of multi-omics has enabled a shift from single-gene associations to systems-level inference. Genomics resolves resistance-conferring variants and copy-number changes; transcriptomics captures inducible responses to chemical exposure; proteomics and metabolomics reveal pathway activity and phenotypic flux. Cross-omics integration, coupled with causal modeling, is beginning to move beyond correlation toward testable mechanisms that explain how network topology and regulatory architecture give rise to resistance in the field.

Translation of these insights into operations is underway. Molecular and metabolic biomarkers are being incorporated into surveillance platforms for risk stratification and early warning. Evidence supports optimization of tool portfolios that combine dual-active ingredient nets, rotations and mixtures for indoor residual spraying, synergist formulations, and novel modes of action with nonchemical measures including Wolbachia deployment, environmental management, and genetic control. Spatiotemporal targeting informed by local resistance ecologies can reduce selection pressure while preserving public-health impact.

Progress now depends on establishing a closed-loop framework that links standardized phenotyping, curated molecular panels, and portable field assays to interoperable data systems, predictive models, and adaptive decision rules with explicit uncertainty quantification and economic evaluation. Prospective, implementation-focused studies should assess effectiveness, cost, and equity under routine program conditions, and data-sharing standards are needed to ensure reproducibility and comparability across geographies. Although the evolutionary contest between mosquitoes and interventions will persist, a coordinated, data-driven approach anchored in multi-omics evidence and operational pragmatism offers a credible path to slowing resistance evolution and safeguarding the long-term effectiveness of vector control.

## Additional Information and Declarations

### Data Availability

The following information was supplied regarding data availability: This is a literature review.
